# FRAILTOOLS study protocol: a comprehensive validation of frailty assessment tools to screen and diagnose frailty in different clinical and social settings and to provide instruments for integrated care in older adults

**DOI:** 10.1186/s12877-019-1042-1

**Published:** 2019-03-18

**Authors:** Marta Checa-López, Myriam Oviedo-Briones, Armando Pardo-Gómez, Jimmy Gonzales-Turín, Tania Guevara-Guevara, José Antonio Carnicero, Shirley Alamo-Ascencio, Francesco Landi, Matteo Cesari, Tomasz Grodzicki, Leocadio Rodriguez-Mañas, on behalf of FRAILTOOLS consortium

**Affiliations:** 1 Fundación de Investigación Biomédica Hospital Universitario de Getafe, Madrid, Spain; 2 Hospital Centro Medicina dell’Invecchiamento, Università Cattolica del Sacro Cuore , Rome, Italy; 3 Fondazione IRCCS Ca’ Granda Ospedale Maggiore Policlinico, University of Milan, Milan, Italy; 4 Jagiellonian University Medical College, Krakow, Poland; 5 Hospital Universitario de Getafe, Madrid, Spain

**Keywords:** Frailty, Assessment tools, Older adults, FRAILTOOLS project

## Abstract

**Background:**

Dozens of scales and questionnaires have been used in the detection of frailty; however, a generalized method for its screening and diagnosis is still lacking in clinical settings. FRAILTOOLS´ main objective is to evaluate the usefulness of frailty scales in the detection of frailty in different clinical and social settings, and its integration in management algorithms for the frail older patient.

**Methods:**

FRAILTOOLS is an observational, longitudinal and prospective study with a follow-up of 6, 12 and 18 months. People older than 75 years old will be recruited from three separate clinical settings (acute geriatric wards, geriatric outpatient clinics and primary care) and one social setting (nursing homes). Exclusion criteria include Mini-mental State Examination < 20 points, and a Barthel index < 90 points, except in nursing home residents (< 40 points). The participants will be recruited in Spain, Italy, France, United Kingdom and Poland. The total sample size will be of 1.940 subjects, 97 subjects in each clinical setting by center. A personal interview with each participant will take place to register data on comorbidity (Charlson Index), functional (SPPB, Barthel and Lawton indexes), cognitive (MMSE) and frailty status (Fried Phenotype, Frailty Trait Scale – short version, SHARE-FI, 35-Items Rockwood Frailty Index, Clinical Frailty Scale, FRAIL scale and Gérontopôle Frailty Screening Tool) in the baseline visit, month 12 and month 18 visit of follow up. At 6 month a phone call will be made to assess whether there have been falls and to check the vital status.

**Discussion:**

Currently, the usefulness of certain assessment tools in social and clinical settings have not been properly assessed, including their ability to predict the individual risk for different adverse outcomes, which is the main interest in daily practice.

The FRAILTOOLS project concentrates on providing screening and diagnostic tools for frailty in those settings where its prevalence is the highest and where efforts in prevention could make a significant change in the trend towards disability.

**Trial registration:**

Comprehensive validation of frailty assessment tools in older adults in different clinical and social settings (FRAILTOOLS), NCT02637518 (date of registration: 12/18/2015).

## Background

From the second half of the twentieth century, life expectancy has increased in developed countries, reaching a mean age of 83 years in countries such as Spain, France and Italy [1, 2]. As a result, Europe is the continent with the highest proportion of older people, but also with a higher dependency ratio rate [2].

From a health and social point of view, the older population has increased requirements, needing specialized care to approach the complexity of their comorbidities [3]. On the other hand, as life expectancy increases, the proportion of older people with any kind of disability rises [4], which in turn increases the need for long term care facilities. All of this leads to additional health and social expenditure [2].

In order to address this situation, new policies are needed, which are particularly aimed at: a) implementing reforms in the pension system, health and care and long-term care facilities, and b) reducing disability and dependency. As to the latter, it is imperative to detect the population at higher risk of disability, with the objective of implementing preventive actions [1, 2].

Within the last 20 years, health and social care professionals dedicated to the field of geriatric medicine have made major efforts in identifying older people requiring specialized attention that may contribute to delay or avoid the appearance of disability. Prior to disability there is an intermediate state known as frailty, a syndrome characterized by diminished capacity to respond to stressors, due to a reduced functional reserve [3]. Frailty is a condition that is closely associated with mortality among community dwelling older adults, followed by organ failure, cancer and terminal dementia [5]. This condition is also related to other adverse outcomes such as: falls, morbidity, disability, polypharmacy, hospitalization, institutionalization and mortality [6].

Multiple studies have been undertaken worldwide to establish the prevalence of frailty [3, 7–9]. The data vary according to countries reaching a percentage of up to 27.3%. A survey of 7510 community-dwelling older adults in 10 European countries found that the prevalence of frailty was higher in southern than in northern Europe [7, 10]. For instance, in Spain the prevalence of frailty was 8.4% among institutionalized and community dwelling persons from rural and urban settings [8]. In Italy, in community-dwelling older adults the frailty prevalence was 13.9% [11]. In the French population older than 55 years-old and free of disability, around 25% are either frail or multimorbid [12]. In the nursing home setting this prevalence doubles in comparison with community dwelling people, reaching 68.8% according to a number of American studies [9, 13]. As frailty is highly associated with age, we should expect an increase in the number of new cases (incidence) of frailty as the European population gets older. Published data ranges from 4% new cases in adults aged over 65 years in Germany to 8% in adults aged over 60 years in Spain after three years of follow-up [14].

Dozens of scales and questionnaires have been used in the detection of frailty [15].; however a universal operational definition of frailty or a generalized method for its screening and diagnosis is still lacking [16]. In different clinical scenarios where the care of the elderly is a priority, such as primary health care or nursing homes, it is imperative to have specific instruments in the detection of frailty according to the characteristics of each level of care.

The FRAILTOOLS Project addresses the needs to validate scales for its application in different clinical and social settings, and its integration in management algorithms for the frail older patient.

## Methods/design

### Objectives

#### Main objective

- Evaluate the usefulness of frailty scales in the detection of frailty in different clinical and social settings.

#### Secondary objectives

- Establish the scale with the highest predictive value according to the most common adverse outcomes in frail patients.
- Design frailty detection algorithms according to the clinical setting.

### Outcomes

#### Primary outcomes

Determine the predictive value of frailty scales according to the adverse outcomes associated with frailty in people older than 75 years old in different settings of clinical care.

- **Mortality:** Data will be obtained from the official registration of the country of the corresponding partner from a participant who does not answer the telephone or who does not have medical follow-up after the last visit.
- **Disability:** It will be defined as a loss of one point in the Short Physical Performance Battery (SPPB), the loss of independence in any Instrumental Activity of Daily Living (IADL) according to the Lawton index, or by a reduction in ≥5 points in the Barthel index [17–19].
- **Falls:** It is an event in which the participant comes to rest inadvertently on the ground or other lower level. Data will be collected by the participant’s verbal recall (self-assessed) and will be registered in the Query-Case Report Form (eCRF).
- **Incident cognitive impairment:** It will be defined by a reduction of ≥2 points in the MMSE [20].

#### Secondary outcomes

- **Performance** of the instruments by clinical setting: seven frailty assessment tools will be used in four different levels of care. The performance of each scale in the classification of frailty will be established.
- **Feasibility** composed by two main conditions: the percentage of people that are assessed by each tool in each setting (adequacy) and the time for carrying out the tool assessment. The utility of both screening and diagnostic tools stems, among other characteristics, from the time needed to pass them. This characteristic is relevant mainly in settings where the demand for attention is high and the time to provide is limited.
- **Sensitivity to change**, one of the problems of many of the tools used to assess frailty is that they have a low sensitivity to change. This is a relevant issue in clinical settings, where monitoring the progress of the patient is of high value. For this purpose, we will evaluate the change in the assessment level of patients observed at 12 and 18 months with each of the tools and their correlations with the changes observed in the functional status of the patients as assessed by SPPB.
- **Qualification as screening and/or diagnosis tool**: evaluate the utility of each scale as a frailty detection method, using pre-established criteria, based on the prevalence of this condition in each setting and the classification performance (Sensitivity, Specificity, Positive and Negative Predictive Values and Likelihood ratios), plus its feasibility, and its sensitivity to change.

### Type of study

This is an observational, longitudinal and prospective study.

### Population

People aged ≥75 years, will be recruited from different clinical and social settings, including acute geriatric wards, geriatric outpatient clinics, primary care centers and nursing homes.

### Inclusion criteria

Participants over 75 years of age, will be consecutively selected, who have signed the informed consent and accepted their voluntary participation.

### Exclusion criteria

- General exclusion criteria are:
  - ○ MMSE score ≤ 20 points.
  - ○ Terminal illness (life expectancy ≤6 months).
- A Barthel Index < 90 in patients from the Acute Geriatric Unit, Outpatient Geriatric Consultation and primary care centers.
- A Barthel Index < 40 in residents from nursing homes.

### Recruitment process and sample size

Prior to the start of the recruitment process, Ethics Committee approval and any other regulatory approvals will be obtained.

Potential candidates will be assessed by geriatricians or other suitably qualified members of the study team and will provide information to each participant about the study and their participation. After each participant has read the participant information sheet and given written informed consent to participate, their eligibility criteria will be assessed and if satisfied, will be enrolled into the study. The participant’s right to decline their participation in the study at any stage without any explanation will be respected. The participant can withdraw the study at any time without giving reasons and with no detriment in their usual medical care. If a participant withdraws from the study after a prior inclusion, this information will be passed immediately to the principal investigator and the procedure for their exclusion will take place.

Each one of the participating centers has a scientific interest in the field of frailty (Table 1). The participants will be recruited in Spain, Italy, France, United Kingdom and Poland.

**Table 1:** Participating centers

Country	Participating center
Spain	Fundación para la Investigación Biomédica del Hospital Universitario de Getafe (FIB-HUG)
Italy	Centro Medicina dell’Invecchiamento (CEMI)/ Università Cattolica del Sacro Cuore
France	Gérontopôle de Toulouse
United Kingdom	Aston University^a^
Poland	Department of Internal Medicine and Gerontology (DIMG) and Division of Internal Medicine of the University Hospital of the JUMC

^a^Aston University replaced Diabetes Frail Limited (DIFRAIL) in United Kindgdom as participating center

Contact with hospitals, primary care and nursing homes will be made through the coordinators of the corresponding Health Centers, requesting the authorization for the recruitment process and signing the proper documents to formalize their participation.

In order to determine the sample size, we have used two assumptions:

1. The sample size is calculated according to the methodology of Peduzzi et al. [21] for a model of 4 variables. The variables included are: age, gender and Charlson index as covariates and frailty status as the main independent variable of interest.
2. The outcomes to be assessed will be death, falls, disability and deterioration in cognitive function. Among these variables, death is the least frequent and generates the highest sample size therefore it can be used for the other three outcomes.
  Although there are some little differences in the mortality rates among the five European countries that participate in this project; the mean mortality rate for people aged ≥75 years in these countries is 10% annually. Therefore, in 18 months it will be 15%, which is the follow-up period forecasted in FRAILTOOLS project.

Within these assumptions the lower limit of the 1-α confidence interval for the accepted number of success is 355 and 388 participants in each setting of care for 95 and 99% CI, respectively. This number must be increased with the forecasted lost to follow-up, in 20%. As a whole, the final sample size is established in 485 persons per setting, which means a final figure of 1940 persons. Thus, every partner will be responsible for the enrolment, assessment and follow-up of 388 older adults (97 per setting).

### Follow-up

The follow-up will be of 18 months as maximum. In order to avoid memory bias, a phone call will be made in month 6 after the inclusion to assess whether there has been a fall or death. At 12 and 18 months, a personal interview with each participant will take place to register data on functional status (SPPB, Barthel and Lawton indexes), cognitive status (MMSE) and frailty status (Fried Frailty Phenotype Criteria, o Frailty Trait Scale – short version, SHARE-FI scale, 35-Items Rockwood Frailty Index, Clinical Frailty Scale, FRAIL scale and Gérontopôle Frailty Screening Tool) (Table 2).

**Table 2:** Flow of the study

Time point	Baseline T1	Follow up		
		Month 6 T2	Month 12 T3	Month 18 T4
ENROLLMENT:				
Eligibility screen	X			
Informed consent	X			
ASSESMENTS:				
Socio-demographic data	X		X	X
Charlson Comorbidity Index	X		X	X
Barthel Index	X		X	X
Lawton Index	X		X	X
SPPB	X		X	X
MMSE	X		X	X
Fried’s Frailty Phenotype Criteria	X		X	X
Frailty Trait Scale – short version	X		X	X
SHARE Frailty Instrument	X		X	X
35-Items Rockwood Frailty index	X		X	X
FRAIL scale	X		X	X
Gérontopôle Frailty Screening Tool	X		X	X
Clinical Frailty Scale	X		X	X
OUTCOMES:				
Disability	X		X	X
Mortality		X	X	X
Falls		X	X	X
Incident cognitive impairment	X		X	X

In case a participant passed away during the follow-up phase of the study, the information will be recorded in the eCRF of the follow-up visits at 6, 12 or 18 months. A document to record death will be filled as an adverse event unrelated to the study. Data regarding mortality will be obtained from the official register of the country of the corresponding partner.

The principal investigator will ensure that the study takes place according to the protocol, to Good Clinical Practice principles, and to the Declaration of Helsinki of 1996. At the baseline visit, frailty assessment tools will be completed. Variables such as socio-demographic data, comorbidities, functional and cognitive status will also be assessed.

The Investigator will ensure that this study is conducted in accordance with the protocol, the principles of the Declaration of Helsinki, International Conference of Harmonization Guidelines for Good Clinical Practice and in full conformity with relevant regulations.

All substantial amendments to the original approved documents will also be sent to the appropriate Ethics Committee and Regulatory Authority (if applicable) for their revision.

The study staff will ensure that the subject’s anonymity is maintained. The subjects will be identified only by a subject code in the eCRF and any electronic database. All documents will be stored securely and only accessible by study staff and authorized personnel. The study will comply with the Data Protection Legislation in each country.

Subjects will not receive any economic compensation for participation in this study.

### Questionnaires and tools assessed

In the Query-Case Report Form the following information will be collected:

- Socio-demographic data: age, gender, race, marital status, education, cohabitation, need for caregiver, setting in which the subject is recruited, country of origin.
- Comorbidities:
  - ○ Charlson Comorbidity Index [22]: This scale predicts the ten-year mortality for a patient by classifying or weighting comorbid conditions. It consists of 19 issues each of which was weighted according to their potential influence on mortality.
- Functional status:
  - ○ Barthel Index [18]: Functional assessment scale that measures the subject’s capacity to perform ten activities of daily living in an independent manner.
  - ○ Lawton Index [17]: Instrument to assess the individual’s capacity to perform IADL independently. These skills are considered more complex than the basic activities of daily living as measured by the Barthel Index.
  - ○ Short Physical Performance Battery [23]: Test designed to measure functional status and physical performance by combining the results of the gait speed, chair stand and balance tests.
- Cognitive status:
  - ○ Mini Mental State Examination – MMSE [20]: It is a widely used test to screen patients for cognitive impairment and to track changes in cognitive functioning over time. It evaluates seven cognitive domains including orientation to time and place, repetition, verbal recall, attention and calculation, language and visual construction.
- Frailty assessment scales:
  - ○ Fried’s Frailty Phenotype Criteria [3]: Well-known scale to diagnose frailty. It is based on the biological causative theory and shows predictive validity for poor health outcomes across a wide range of illnesses and procedures. This tool combines a total of five variables, three of them self-referred: weight loss, exhaustion and reduced physical activity; and other two objective variables: weakness assessed by grip strength, and slowness measured by gait speed.
  - ○ Frailty Trait Scale – short version [24]: Scale that emerged from a population study in Toledo, Spain. It evaluates three dimensions: nutrition (BMI), physical activity (PASE), and nervous system (balance test).
  - ○ SHARE Frailty Instrument (6,7): Screening instrument for frailty proposed for the primary health care setting. It explores five dimensions: exhaustion, loss of appetite, weakness measured by grip strength, difficulty walking and low physical activity.
  - ○ 35-Items Rockwood Frailty index [25]: Scale with 35 items, based on data routinely collected as part of a geriatric assessment. It includes items on chronic diseases, basic and instrumental disabilities in activities of daily living, serum vitamin D, cognition, physical performance, nutrition, visual and hearing impairment.
  - ○ FRAIL scale [26]: Self-assessed short questionnaire, taking into account five different aspects: Fatigue, Resistance, Ambulation, Illness and Loss of weight. It does not require measurements or administration by healthcare professionals.
  - ○ Gérontopôle Frailty Screening Tool [27]: It is a short questionnaire addressed to primary care physicians with a total of six questions assessing the individual’s social, physical, functional and cognitive situation. There is no clear cut-off point to classify the patient as frail or not.
  - ○ Clinical Frailty Scale o Rockwood modified [28]: Scale that uses clinical descriptors and pictographs to stratify older adults according to level of vulnerability. It mixes items such as comorbidity, cognitive impairment and disability. According to the authors, it provides predictive information about mortality or institutionalization similar to that of other established tools.

The study is ongoing. Follow-up visits finished on November 30th. Statistical analysis will be run until April 1st, 2019. The publication of the full results will take place on the second half of 2019.

### Statistical analysis

FRAILTOOLS aims to 1) assess the selected instruments (scales) for both screening and diagnosis purposes in each of the four settings: acute geriatric wards, geriatric outpatient clinics, primary care centers and nursing homes; and 2) explore the sequential or consecutive assessment of the older population amongst settings.

Regarding the first objective of the analysis, we will assess the associations of each of the seven scales with the outcome for each setting and outcome through logistic regressions using age, sex and Charlson index as possible confounders. With these variables, we will construct three logistic regression models: unadjusted, adjusted by age and gender, and adjusted by age, gender and Charlson Index. Firstly, we will compute the classification performance (sensitivity, specificity, ROC curve, AUC, predictive values, likelihood ratios) for each model. This way, we will assess the most accurate scale for both screening (the most sensitive tool) and diagnostic purposes (the most specific tool), for each setting and for each outcome. Secondly, we will study the feasibility of the models, considering the time needed for the scale and the percentage of patients that can be evaluated per case.

Combining both outcomes by creating an index (classification performance * proportion of evaluated patients), we will determine the best scale (maximum value of the index) per setting for both screening and diagnosis. Thirdly, we will evaluate the sensitivity to change of the scales and the covariance of the scales with other measures as the SPPB through a mixed linear model.

For the second objective, we will first analyze the external validity of each model by using the data of each setting in the other models during the last phase of the project. Afterwards, we will evaluate which set of instruments is more appropriate for sequential or consecutive assessment when patients move between different settings: e.g. from nursing homes to geriatrics wards.

## Discussion

Frailty is the main risk factor for the appearance of disability [29]; once disability arises, recovery is unlikely [30]. Frailty can be reversed spontaneously [29, 31] or by exercise-based interventions [32].

In view of its prognostic power to cause disability, its high prevalence and potential reversibility, frailty is the ideal objective to approach the disability challenge in our elders [29, 31].

Many studies have demonstrated the utility of certain assessment tools to evaluate frailty in populations, however, the individual risk for disability has not been properly evaluated, which is the main interest in the daily clinical and social settings [14, 33]. Indeed, so far, no published studies have validated the usefulness and feasibility of frailty tools in geriatric units, primary care centers and nursing homes; where the prevalence of people with frailty is high and the risk to develop disability is palpable. FRAILTOOLS study will contribute to validate tools to screen and diagnose frailty in different clinical scenarios; with the purpose of implementing it in daily practice and creating diagnostic algorithms according to the setting assessed.

Therefore, it can be said that the FRAILTOOLS project is an *original* initiative; *relevant*, by focusing its efforts on the main risk factor for disability; *pertinent*, concentrating on providing screening and diagnostic tools for frailty in settings where its prevalence is the highest and where efforts in prevention could make a significant change in the trend towards disability. FRAILTOOLS also contributes to the initiative of the European’s Commission on Frailty, Integrated care and multi-chronic conditions by: 1) the application of coordinated and innovative preventive measures, 2) development and implantation of screening and early diagnostic programs of frailty, which include the optimization of functional capacity and the development of guidelines to manage frailty, 3) improve healthcare systems, 4) implement health promotion strategies, and 5) facilitate the exchange of Good Clinical Practice and expanding knowledge networks, and 6) promote innovation in healthcare at European level.

## Abbreviations

AUC: Area Under the Curve
BMI: Body Mass Index
eCRF: Query-Case Report Form
FIB-HUG: Fundación para la Investigación Biomédica del Hospital Universitario de Getafe
IADL: Instrumental Activities of Daily Living
